# Supplementary material for: Solanum tuberosum Microtuber Development under Darkness Unveiled through RNAseq Transcriptomic Analysis
Source: Int J Mol Sci. 2022 Nov 10;23(22):13835. doi: 10.3390/ijms232213835 (PMC9696990; doi:10.3390/ijms232213835)
Supplement: Supplementary file 1 [file ijms-23-13835-s001.zip › ijms-1961805-supplementary Figure S1.pdf]

International Journal of
Molecular Sciences

MDPI

Article

Supplementary Figures

Solanum tuberosum Microtuber development under darkness unveiled through RNAseq Transcriptomic analysis

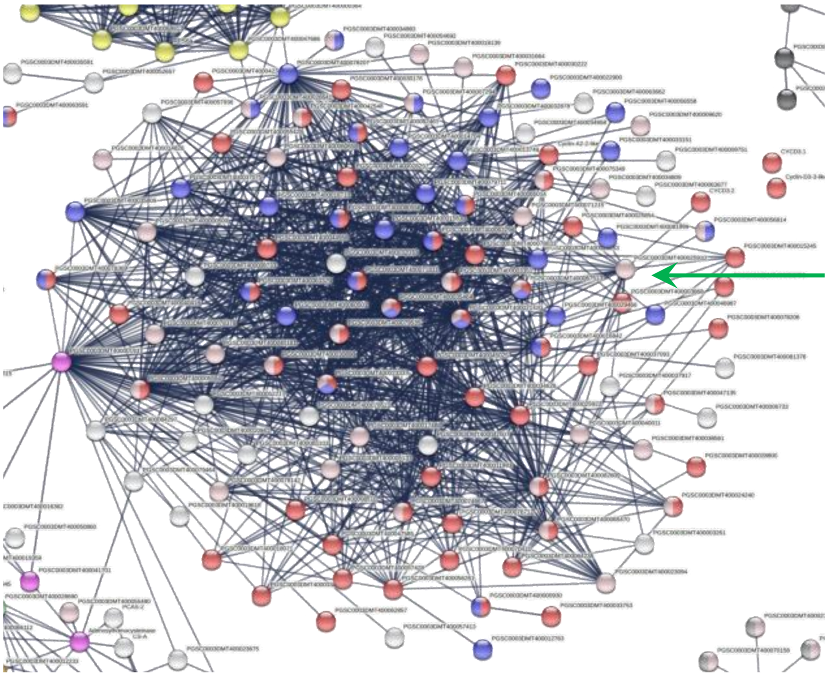

Figure S1. CC PPI network of upregulated DEG derived using STRING database from genome-wide analysis of potato *S. tuberosum* with highest confidence (0.860). Selected proteins are highlighted with the name of the gene.

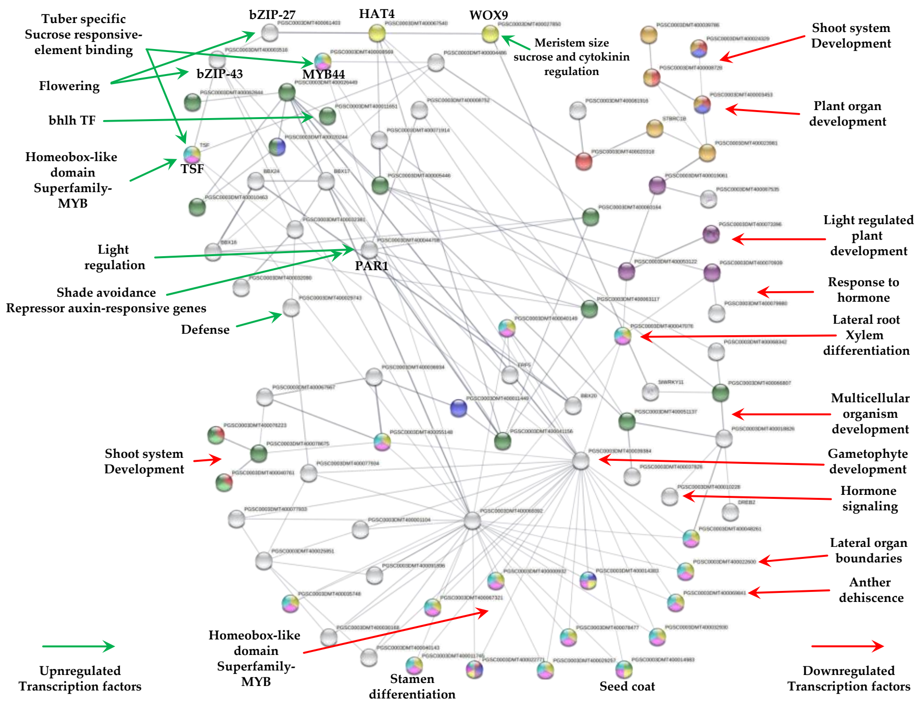

Figure S2. PPI network of up and downregulated TFs derived using STRING database from genome-wide analysis of potato *S. tuberosum* with medium confidence (0.400). The figure represents a full network, the edges indicate both functional and physical protein associations.
