# Supplementary material for: Solanum tuberosum Microtuber Development under Darkness Unveiled through RNAseq Transcriptomic Analysis
Source: Int J Mol Sci. 2022 Nov 10;23(22):13835. doi: 10.3390/ijms232213835 (PMC9696990; doi:10.3390/ijms232213835)
Supplement: Supplementary file 1 [file ijms-23-13835-s001.zip › ijms-1961805-supplementary Table S1.pdf]

International Journal of *Molecular Sciences*

MDPI

Article

Supplementary Tables S1

Solanum tuberosum Microtuber development under darkness unveiled through RNAseq Transcriptomic analysis.

Valencia-Lozano E, Herrera-Isidron L, Flores-López JA, Recoder-Melendez OS, Barraza-Celis A and Cabrera-Ponce JL

Tables S1. Upregulated Genes with Response to cytokinins and Associated function,

Response to Osmotic stress and Associated function, Response to One Carbon Metabolism and Associated function, Response to Carbon Metabolism and Associated function, Related to Citrate Cycle and Associated function, to Acyl Carrier Proteins and Associated function, to Fatty acid biosynthesis and metabolism and Associated function, to Thylakoids and Associated function, to Redoxins and Associated function, to Response to oxidative stress and Associated function, to Ribosomal proteins, Mutants and Associated function, to Immunophylins and Associated function, to Mitotic cell cycle and Associated function, to E2F activation and Associated function, to Cytoskeleton and Associated function, to Motor proteins and Associated function, to Methylation and Associated function, to Histone binding and Associated function, to Sterol biosynthetic process and terpenoid backbone biosynthesis and Associated function, to Amino sugar and nucleotide sugar metabolism and Associated function, to Sulfur metabolism and Associated function, to Disulfide isomerase activity and Associated function, Related to DNA replication and Associated function. Primer design of DEG used to validate the genome-wide analysis of MTs development of potato S. tuberosum cv. Alpha.

Upregulated Genes with Response to cytokinins and Associated function.

N°	ID *A. thaliana*	ID *Solanum tuberosum*	Associated function
1	*ARR8*	PGSC0003DMT400076758	Two-component response regulator arr-a family.
2	*ARR1*	PGSC0003DMT400060506	Two-component response regulator arr-b family.
3	*ARR2*	PGSC0003DMT400065835	Two-component response regulator arr-b family.
4	*RR3*	PGSC0003DMT400076758	Functions as response regulator involved in His-to-Asp phosphorelay signal transduction.
5	*ARR4*	PGSC0003DMT400058306	Two-component response regulator arr-a family.
6	*ARR5*	PGSC0003DMT400063187	Two-component response regulator ARR5.
7	*ARR9*	PGSC0003DMT400076726	Two-component response regulator ARR9.
8	*ARR11*	PGSC0003DMT400031260	Two-component response regulator ARR11.
9	*FBA6*	PGSC0003DMT400006894	Fructose-bisphosphate aldolase, class I.

10	*ENO1*	PGSC0003DMT400062986	Enolase 1, chloroplastic.
11	*EBP*	PGSC0003DMT400043311	Ethylene-responsive transcription factor RAP2-3.
12	*GAPCP-1*	PGSC0003DMT400029242	Glyceraldehyde-3-phosphate dehydrogenase GAPCP1, chloroplastic.
13	*DAP*	PGSC0003DMT400069487	Pyridoxal phosphate (PLP)-dependent transferases superfamily protein.
14	*ACC1*	PGSC0003DMT400022054	Acetyl-Coa carboxylase/ biotin carboxylase 1.
15	*TIL*	PGSC0003DMT400020425	Apolipoprotein d and lipocalin family protein.
16	*TPI*	PGSC0003DMT400071330	Triosephosphate isomerase, cytosolic.
17	*MDH*	PGSC0003DMT400050235	Plastidic nad-dependent malate dehydrogenase.
18	*APX3*	PGSC0003DMT400015799	L-ascorbate peroxidase 3.
19	*APS1*	PGSC0003DMT400034962	3'-phosphoadenosine 5'-phosphosulfate synthase.
20	*CSP41A*	PGSC0003DMT400033560	Chloroplast stem-loop binding protein of 41 kDa a, chloroplastic.
21	*ZFP5*	PGSC0003DMT400040327	Zinc finger protein 5.
22	*AT5G19370*	PGSC0003DMT400012014	Rhodanese-like domain-containing protein.
23	*AT1G68360*	-	Diaminopimelate epimerase, chloroplastic.
24	*LOGL10*	PGSC0003DMT400057413	Riboside 5'-monophosphate phosphoribohydrolase logl10.
25	*LOG3*	PGSC0003DMT400027157	Cytokinin riboside 5'-monophosphate phosphoribohydrolase.
26	*HXK1*	PGSC0003DMT400006474	Hexokinase-1.

Upregulated Genes with Response to Osmotic stress and Associated function.

**N°**	**ID *A. thaliana***	**ID *Solanum tuberosum***	**Associated function**
1	*FBA6*	PGSC0003DMT400006894	Fructose-bisphosphate aldolase, class I.
2	*CRT1b*	Calreticulin	Calreticulin 1b.
3	*TUB8*	PGSC0003DMT400028800	Tubulin beta-8 chain.
4	*LOS2*	Enolase-like	Bifunctional enolase 2/ transcriptional activator.
5	*LOS2*	PGSC0003DMT400028690	Bifunctional enolase 2/ transcriptional activator.
6	*ENO1*	PGSC0003DMT400062986	Enolase 1, chloroplastic.
7	*TPI*	PGSC0003DMT400071330	Triosephosphate isomerase, cytosolic.
8	*mMDH1*	MMDH	Lactate/malate dehydrogenase family protein.
9	*MDH*	PGSC0003DMT400050235	Plastidic nad-dependent malate dehydrogenase.
10	*FSD2*	PGSC0003DMT400013447	Superoxide dismutase [Fe] 2, chloroplastic.
11	*FSD2*	PGSC0003DMT400070920	Superoxide dismutase [Fe] 2, chloroplastic.
12	*MSD1*	PGSC0003DMT400042937	Superoxide dismutase [Mn] 1, mitochondrial.
13	*AT3G52960*	Peroxiredoxin-2E-2	Peroxiredoxin-2E, chloroplastic.
14	*At5g06290*	PGSC0003DMT400042222	2-Cys peroxiredoxin BAS1-like, chloroplastic.
15	*GPX7*	PGSC0003DMT400067601	Putative glutathione peroxidase 7, chloroplastic.
16	*AT3G52960*	PGSC0003DMT400072641	Peroxiredoxin-2E.
17	*At5g06290*	PGSC0003DMT400072569	2-Cys peroxiredoxin BAS1-like, chloroplastic.
18	*GSTF8*	PGSC0003DMT400029561	Glutathione S-transferase F8, chloroplastic.

19	*CNX1*	PGSC0003DMT400036920	Calnexin homolog 1.
20	*ATARCA*	PGSC0003DMT400014419	Transducin/WD40 repeat-like superfamily protein.
21	*RACK1B_AT*	PGSC0003DMT400074094	Receptor for activated C kinase 1B.

Upregulated Genes with Response to One Carbon Metabolism and Associated function.

N°	ID *A. thaliana*	ID *Solanum tuberosum*	Associated function
1	*SHM1*	PGSC0003DMT400016362	Serine hydroxymethyltransferase 1, mitochondrial.
2	*THY-1*	PGSC0003DMT400001937	Bifunctional dihydrofolate reductase-thymidylate synthase 1.
3	*MAT3*	PGSC0003DMT400072701	Methionine adenosyltransferase 3.
4	*SMT2*	PGSC0003DMT400047969	24-methylenesterol C-methyltransferase 2.
5	*SHM4*	PGSC0003DMT400040717	Glycine hydroxymethyltransferase.

Upregulated Genes with Response to Carbon Metabolism and Associated function.

N°	ID *A. thaliana*	ID *Solanum tuberosum*	Associated function
1	*CAC2*	PGSC0003DMT400042238	Acetyl Co-enzyme a carboxylase biotin carboxylase subunit.
2	*CAC1*	PGSC0003DMT400083101	Biotin carboxyl carrier protein of acetyl-CoA carboxylase 1.
3	*ACAT2*	PGSC0003DMT400028289	Acetyl-CoA acetyltransferase, cytosolic 1.
4	*EMB3003*	PGSC0003DMT400023831	Pyruvate dehydrogenase e2 component.
5	*AT2G35120*	PGSC0003DMT400027015	Glycine cleavage system H protein 2, mitochondrial.

6	*At1g01090*	PGSC0003DMT400007571	Pyruvate dehydrogenase E1 component subunit alpha-3, chloroplastic.
7	*SHM4*	PGSC0003DMT400040717	Glycine hydroxymethyltransferase.
8	*SHM1*	PGSC0003DMT400016362	Serine hydroxymethyltransferase 1.
9	*MTHFR2*	PGSC0003DMT400041701	Methylenetetrahydrofolate reductase (nadph).
10	*ASP1*	PGSC0003DMT400017112	Aspartate aminotransferase, mitochondrial.
11	*PPC3*	PGSC0003DMT400054791	Phosphoenolpyruvate carboxylase (PEPC).
12	*FDH*	PGSC0003DMT400001303	Formate dehydrogenase, chloroplastic/mitochondrial.
13	*AT2G34590*	PGSC0003DMT400019358	Pyruvate dehydrogenase E1 component subunit beta-3, chloroplastic.
14	*PPC3*	PGSC0003DMT400039809	Phosphoenolpyruvate carboxylase (PEPC).
15	*mMDH1*	PGSC0003DMT400032266	Lactate/malate dehydrogenase family protein.
16	*PPC4*	PGSC0003DMT400019322	Phosphoenolpyruvate carboxylase 4.
17	*MDH*	PGSC0003DMT400050235	Plastidic nad-dependent malate dehydrogenase.
18	*PKP-ALPHA*	PGSC0003DMT400066112	Plastidial pyruvate kinase 1.
19	*PKp3*	PGSC0003DMT400062238	Plastidial pyruvate kinase 3, chloroplastic.
20	*AT3G52990*	PGSC0003DMT400072411	Pyruvate kinase family protein.
21	*AT3G02360*	PGSC0003DMT400064670	6-phosphogluconate dehydrogenase, decarboxylating 3.
22	*EMB2024*	PGSC0003DMT400047627	Probable 6-phosphogluconolactonase 5, chloroplastic.
23	*AT3G52990*	PGSC0003DMT400006945	Pyruvate kinase family protein.
24	*GAPCP-2*	PGSC0003DMT400029242	Glyceraldehyde-3-phosphate dehydrogenase GAPCP2.

25	*GAPC2*	PGSC0003DMT400044944	Glyceraldehyde-3-phosphate dehydrogenase GAPC2.
26	*PGK*	PGSC0003DMT400056871	Encodes cytosolic phosphoglycerate kinase (PGK)
27	*AT1G12230*	PGSC0003DMT400012218	Aldolase superfamily protein.
28	*FBA6*	PGSC0003DMT400006894	Fructose-bisphosphate aldolase, class I.
29	*LOS2*	PGSC0003DMT400007048	Bifunctional enolase 2/ transcriptional activator.
30	*ENO1*	PGSC0003DMT400062986	Enolase 1, chloroplastic.
31	*TPI*	PGSC0003DMT400071330	Triosephosphate isomerase, cytosolic.
32	*OASA1*	PGSC0003DMT400082113	O-acetylserine (thiol) lyase (oas-tl) isoform a1.
33	*SERAT2;2*	PGSC0003DMT400037270	Serine acetyltransferase 3, mitochondrial.
34	*AT1G22170*	PGSC0003DMT400024493	Phosphoglycerate mutase family protein.
35	*LOS2*	PGSC0003DMT400028690	Bifunctional enolase 2/ transcriptional activator.
36	*CYSC1*	PGSC0003DMT400000156	Bifunctional L-3-cyanoalanine synthase/cysteine synthase C1, mitochondrial.
37	*PGDH*	PGSC0003DMT400023675	D-3-phosphoglycerate dehydrogenase / 2-oxoglutarate reductase.
38	*EDA9*	PGSC0003DMT400059818	D-3-phosphoglycerate dehydrogenase / 2-oxoglutarate reductase.

Upregulated Genes Related to Citrate Cycle and Associated function.

**N°**	**ID *A. thaliana***	**ID *Solanum tuberosum***	**Associated function**
1	*ACLB-1*	PGSC0003DMT400061710	ATP-citrate synthase beta chain protein 1.
2	*PPC3*	PGSC0003DMT400054791	Cytosolic phosphoenolpyruvate carboxylase (PEPC).

3	*PPC3*	PGSC0003DMT400039809	Cytosolic phosphoenolpyruvate carboxylase (PEPC).
4	*ACLB-2*	PGSC0003DMT400011963	ATP-citrate synthase beta chain protein 2.
5	*FDH*	PGSC0003DMT400001303	Formate dehydrogenase.
6	*mMDH1*	PGSC0003DMT400032266	Lactate/malate dehydrogenase family protein.
7	*PPC4*	PGSC0003DMT400019322	Phosphoenolpyruvate carboxylase 4.
8	*ACLA-2*	PGSC0003DMT400035064	ATP-citrate synthase alpha chain protein 2.
9	*At1g01090*	PGSC0003DMT400007571	Pyruvate dehydrogenase E1 component subunit alpha 3.
10	*MDH*	PGSC0003DMT400050235	Plastidic nad-dependent malate dehydrogenase.
11	*PKP-ALPHA*	PGSC0003DMT400066112	Plastidial pyruvate kinase 1.
12	*AT2G34590*	PGSC0003DMT400019358	Pyruvate dehydrogenase E1 component subunit beta-3.
13	*AT3G52990*	PGSC0003DMT400006945	Pyruvate kinase family protein.
14	*AT3G52990*	PGSC0003DMT400072411	Pyruvate kinase family protein.
15	*AT4G17260*	PGSC0003DMT400012233	Lactate/malate dehydrogenase family protein.
16	*EMB3003*	PGSC0003DMT400023831	Pyruvate dehydrogenase e2 component.

Upregulated Genes Related to Acyl Carrier Proteins and Associated function.

**N°**	**ID *A. thaliana***	**ID *Solanum tuberosum***	**Associated function**
1	*ACP4*	PGSC0003DMT400036981	Acyl carrier protein 4.
2	*ACP2*	PGSC0003DMT400079040	Acyl carrier protein 2.
3	*ACP5*	PGSC0003DMT400027652	Acyl carrier protein 5.

4	*AT5G10160*	PGSC0003DMT400032718	Thioesterase superfamily protein.
5	*EMB3147*	PGSC0003DMT400055055	Catalytics transferases acyl-carrier-protein S-malonyltransferases.
6	*AT1G24360*	PGSC0003DMT400069374	3-oxoacyl-[acyl-carrier-protein] reductase.

Upregulated Genes Related to Fatty acid biosynthesis and metabolism and Associated function.

**N°**	**ID *A. thaliana***	**ID *Solanum tuberosum***	**Associated function**
1	*AT5G59770*	PGSC0003DMT400040154	Very-long-chain (3R)-3-hydroxyacyl-CoA dehydratase.
2	*KCR1*	PGSC0003DMT400032914	Very-long-chain 3-oxoacyl-CoA reductase 1.
3	*CER10*	PGSC0003DMT400060224	3-oxo-5-alpha-steroid 4-dehydrogenase family protein.
4	*PAS2*	PGSC0003DMT400060612	Very-long-chain (3r)-3-hydroxyacyl-coa dehydratase.
5	*AT5G10160*	PGSC0003DMT400032718	Thioesterase superfamily protein.
6	*AT1G24360*	PGSC0003DMT400045027	3-oxoacyl-[acyl-carrier-protein] reductase, chloroplastic.
7	*FAB1*	PGSC0003DMT400035344	3-oxoacyl-[acyl-carrier-protein] synthase II, chloroplastic.
8	*T3P18.20*	PGSC0003DMT400067652	3-oxoacyl-[acyl-carrier-protein] synthase III, chloroplastic.
9	*KASI*	PGSC0003DMT400077561	3-oxoacyl-[acyl-carrier-protein] synthase I, chloroplastic.
10	*AT1G24360*	PGSC0003DMT400069374	3-oxoacyl-[acyl-carrier-protein] reductase, chloroplastic.
11	*CAC1*	PGSC0003DMT400083101	Biotin carboxyl carrier protein of acetyl-CoA carboxylase 1, chloroplastic.

12	*EMB3147*	PGSC0003DMT400055055	Catalytics transferases [acyl-carrier-protein] S-malonyltransferases binding.
13	*CAC2*	PGSC0003DMT400042238	Acetyl Co-enzyme a carboxylase biotin carboxylase subunit.
14	*ACAT2*	PGSC0003DMT400028289	Acetyl-CoA acetyltransferase, cytosolic 1.

Upregulated Genes Related to Thylakoids and Associated function.

**N°**	**ID *A. thaliana***	**ID *Solanum tuberosum***	**Associated function**
1	*FDH*	PGSC0003DMT400001303	Formate dehydrogenase, chloroplastic/mitochondrial.
2	*AT2G34460*	PGSC0003DMT400021415	NAD(P)-binding Rossmann-fold superfamily protein.
3	*AOR*	PGSC0003DMT400079606	Oxidoreductase, zinc-binding dehydrogenase family protein.
4	*PnsB5*	PGSC0003DMT400015589	Photosynthetic ndh subcomplex b5.
5	*PSI-P*	PGSC0003DMT400029398	Thylakoid membrane phosphoprotein of 14 kda.
6	*CSP41A*	PGSC0003DMT400033560	Chloroplast stem-loop binding protein of 41 kDa a, chloroplastic.
7	*ATHM2*	PGSC0003DMT400055831	Thioredoxin superfamily protein.
8	*AT3G52960*	PGSC0003DMT400072641	Peroxiredoxin-2E, chloroplastic.
9	*AT3G52960*	PGSC0003DMT400007049	Peroxiredoxin-2E-2, chloroplastic-like.
10	*FSD2*	PGSC0003DMT400013447	Superoxide dismutase [Fe] 2, chloroplastic.
11	*FSD2*	PGSC0003DMT400070920	Superoxide dismutase [fe].
12	*TROL*	PGSC0003DMT400000605	Rhodanese-like domain-containing protein 4.

13	*Pnsl5*	PGSC0003DMT400013412	Peptidyl-prolyl cis-trans isomerase b (cyclophilin b).

Upregulated Genes Related to Redoxins and Associated function.

N°	ID *A. thaliana*	ID *Solanum tuberosum*	Associated function
1	*GPX7*	PGSC0003DMT400067601	Putative glutathione peroxidase 7, chloroplastic.
2	*MSD1*	PGSC0003DMT400042937	Superoxide dismutase [Mn] 1, mitochondrial.
3	*At5g06290*	PGSC0003DMT400072569	2-Cys peroxiredoxin BAS1-like.
4	*ATHM2*	PGSC0003DMT400055831	Thioredoxin superfamily protein.
5	*AT3G52960*	PGSC0003DMT400072641	Peroxiredoxin-2E, chloroplastic.
6	*AT3G52960*	PGSC0003DMT400007049	Peroxiredoxin-2E-2, chloroplastic-like.
7	*FSD2*	PGSC0003DMT400013447	Superoxide dismutase [Fe] 2, chloroplastic.
8	*FSD2*	PGSC0003DMT400070920	Superoxide dismutase [fe], chloroplastic.
9	*At5g06290*	PGSC0003DMT400042222	2-Cys peroxiredoxin BAS1-like, chloroplastic.

Upregulated Genes Related to Response to oxidative stress and Associated function.

N°	ID *A. thaliana*	ID *Solanum tuberosum*	Associated function
1	*GPX7*	PGSC0003DMT400067601	Putative glutathione peroxidase 7, chloroplastic.
2	*MSD1*	PGSC0003DMT400042937	Superoxide dismutase [Mn] 1, mitochondrial.
3	*At5g06290*	PGSC0003DMT400072569	2-Cys peroxiredoxin BAS1-like, chloroplastic.
4	*AT3G52960*	PGSC0003DMT400072641	Peroxiredoxin-2E, chloroplastic.

5	*AT3G52960*	PGSC0003DMT400007049	Peroxiredoxin-2E-2, chloroplastic-like.
6	*FSD2*	PGSC0003DMT400013447	Superoxide dismutase [Fe] 2, chloroplastic.
7	*FSD2*	PGSC0003DMT400070920	Superoxide dismutase [fe], chloroplastic.
8	*At5g06290*	PGSC0003DMT400042222	2-Cys peroxiredoxin BAS1-like, chloroplastic.

International Journal of *Molecular Sciences*

Upregulated Genes Related to Ribosomal proteins, Mutants and Associated function. 1

N°	ID *A. thaliana*	ID *S. tuberosum*	Annotated function	Extrarribosomal function	Mutant phenotype
1	*RPSA*	PGSC0003DMT400039497	Ribosomal protein SA	-	Cases of isolated congenital asplenia.
2	*RPS2e*	PGSC0003DMT400063904	Ribosomal protein S2e	-	-
3	*RPS3a*	PGSC0003DMT400083968	Ribosomal protein S3a	-	-
4	*RPS6a*	RPS6a	Ribosomal protein S6a	Key downstream effector of the TOR signaling pathway (Chen et al., 2018 [1]). Free RPS6 binds directly to the rRNA gene promoter in Arabidopsis (Kim et al., 2014 [2])	Embryo lethal (Tzafrir et al, 2003 [3]); Reduced leaf area and enhanced var2-mediated leaf variegation (Wang et al., 2018 [4]), slow growth (haplodeficiency) in paralog A-B double mutant (Creff et al., 2010 [5]). Low temperature albino phenotype (Wang et al., 2018 [4]), pale leaves and defective thylakoid architecture (Sun et al., 2016 [6]).
5	*RPS13*	PGSC0003DMT400070434	Ribosomal protein S2e	Induced by low temperature treatment (Kim et al., 2004 [7]).	Narrow pointed leaves; change of shape of trichomes; delayed root growth (Ito et al., 2000 [8]); late flowering, Embryo lethal (Romani et al., 2012 [9]).
6	*RPS15a-1*	PGSC0003DMT400065156	Ribosomal protein S15a	-	Type II uS8, evolutionarily divergent and plant-specific paralogue, larger leaf, root and cell area (Szick-Miranda et al., 2010 [10])
7	*RPS16*	PGSC0003DMT400077257	Ribosomal protein S9	-	Abnormal pollen development and impaired pollen tube growth (Lu et al., 2020 [11]). Embryo lethal (Ma and Dooner, 2004 [12]), albino in three-leaf stage in rice (Qiu et al., 2018 [13]).

8	*RPS17*	PGSC0003DMT400060127	Ribosomal protein S17	-	Embryo lethal (Tzafrir et al., 2004 [14]); Reductions in growth, leaf pigments and photosynthesis (Romani et al., 2012 [9]), embryo lethality in maize (Schultes et al., 2000 [15]).
9	*RPS25e*	PGSC0003DMT400054605	Ribosomal protein S25e	-	-
10	*RPS29*	PGSC0003DMT400069470	Ribosomal protein S29	Contain the conserved zinc finger motif that could take on a wide variety of cellular functions by providing stable structural scaffolds (Chan et al., 1993 [16]).	-
11	*RPS30*	PGSC0003DMT400006164	Ribosomal protein S30	-	Indole-3-carbinol tolerance (Finkelshtein et al., 2021 [17]).
12	*RPL4*	PGSC0003DMT400071725	Ribosomal protein L1	-	Embryo lethal. Abnormal basal ribosome activity, abnormal transition from globular to heart stage of embryogenesis (Romani et al., 2012 [9]).
13	*RPL8*	PGSC0003DMT400064385	Ribosomal protein L8	-	Embryo lethal (Tzafrir et al., 2004 [14]).
14	*RPL11*	PGSC0003DMT400031869	Ribosomal protein L11	-	Mutant has decreased the effective quantum yield of photosystem II; Pale green plants; Reduced growth rate.
15	*RPL12*	PGSC0003DMT400008687	Ribosomal protein L12	-	Decreased non-host disease resistance against bacterial pathogens (Nagaraj et al., 2016 [18]); Albino lethal phenotype at seedling stage (Zhao et al., 2016 [19]).
16	*RPL13a*	PGSC0003DMT400040760	Ribosomal protein L13a	Overexpression of a brinjal RPL13a in potato has been observed to enhance resistance to Verticillium dahliae (Yang et al., 2013 [20]).	Single base substitution affects chloroplast development in rice grown under low temperature conditions (Song et al., 2014 [21]),

					lethal albino seedlings of T-DNA insertion mutants (Lee et al., 2019 [22]).
17	*RPL18*	PGSC0003DMT400011931	Ribosomal protein L18	-	Non-cellularized endosperm and arrested embryos (Zhang et al., 2015 [23]); Reduced leaf area (Wang et al., 2018 [4]); Required for both male gametophyte function and embryo development (Yan et al., 2016 [24]).
18	*RPL24*	PGSC0003DMT400085766	Ribosomal protein L24	Essential for translation initiation of several proteins, including ETTIN (ETT) and MONOP-TEROS (MP) (auxin response factors) (Nishimura et al., 2005 [25]); Plays an indirect role in promoting MIR gene transcription (Li et al., 2017 [26]).	Suppresses proline accumulation of the parental mutant zinc ring finger 1 (atrzf1) of *Arabidopsis thaliana* (Park et al., 2017 [27]); Reductions in growth, leaf pigments and photosynthesis (Romani et al., 2012 [9]).
19	*RPL24e*	PGSC0003DMT400072655	Ribosomal protein L24e	-	Narrow pointed leaves; cotyledon and leaf vascular pattern defects; defects of the apical-basal pattern of the gynoecium; reduced plant size; reduced fertility (Nishimura et al., 2005 [25]).
20	*RPL25*	PGSC0003DMT400040794	Ribosomal protein L25	-	-
21	*RPL27*	RPL27	Structural constituent of the ribosome; in-volved in translation	-	Abnormal basal ribosome activity, abnormal transition from globular to heart stage of embryogenesis.
22	*RPL27*	PGSC0003DMT400061374	Ribosomal protein L27	-	Abnormal basal ribosome activity, abnormal transition from globular to heart stage of embryogenesis.
23	*RPL27a*	PGSC0003DMT400046820	Ribosomal protein L27a	-	Affected plant shoot development, including leaf pattern, inflorescence and floral meristem function, and seed development (Szakonyi et al., 2011 [28]). Affectation of the development of the female gametophyte (Zsögön et al., 2014 [29]).

24	*RPL29e*	PGSC0003DMT400069847	Ribosomal protein L29e	-	Osteogenesis delayed and fragility of adult bone increased (Oristian et al., 2009 [30]).
25	*RPL31*	PGSC0003DMT400009481	Ribosomal protein L31	-	Slow growth phenotype (Aseev et al., 2011 [31]).
26	*RPL34e*	PGSC0003DMT400080291	Ribosomal protein L34e	-	-
27	*RPL35*	PGSC0003DMT400060449	Ribosomal protein L35	-	Affectation of the development of the female gametophyte (Zsögön et al., 2014 [29]). Abnormal basal ribosome activity, abnormal transition from globular to heart stage of embryogenesis (Romani et al., 2012 [9]).
28	*RPL35*	PGSC0003DMT400069913	Ribosomal protein L35	-	-
29	*RPL40*	PGSC0003DMT400047686	Ribosomal protein L40	Formation of a special cellular structure to facilitate prerRNA processing and ribosome assembly (Finley et al., 1989 [32]).	Embryo lethal (Tzafrir et al., 2004 [14]).
30	*RPL40*	PGSC0003DMT400000364	Ribosomal protein L40	The covalent association of these RPs to ubiquitin could promote the formation of a special cellular structure to facilitate prerRNA processing and ribosome assembly (Finley et al., 1989 [32]).	Embryo lethal (Tzafrir et al., 2004 [14]).
31	*ATARCA*	PGSC0003DMT400014419	Transducin/WD40 repeat-like superfamily protein; Major component of the RACK1.	Key integrator and mediator of hormonal control of translation (Chen et al., 2006 [33]; Guo and Chen, 2008 [34]); connects heterotrimeric G protein with a MAPK cascade to form a unique signaling pathway in plant immunity (Cheng et al., 2015 [35])	Pointed leaf phenotype and partial genetic redundancy of paralogs by complementation studies (Guo and Chen, 2008 [34]), AtRPL4D restored by sac52-d (AtRPL10A), sac53-d (AtRACK1A), sac56-d, and thermospermine (Kakehi et al., 2015 [36]).

32	*RACK1*	PGSC0003DMT400074094	Receptor for activated C kinase 1B	Key integrator and mediator of hormonal control of translation (Chen et al., 2006 [33]; Guo and Chen, 2008 [34]); connects heterotrimeric G protein with a MAPK cascade to form a unique signaling pathway in plant immunity (Cheng et al., 2015 [35])	Pointed leaf phenotype and partial genetic redundancy of paralogs by complementation studies (Guo and Chen, 2008 [34]), AtRPL4D restored by sac52-d (AtRPL10A), sac53-d (AtRACK1A), sac56-d, and thermospermine (Kakehi et al., 2015 [36]).

2

International Journal of *Molecular Sciences*

MDPI

Upregulated Genes Related to Immunophylins and Associated function.

**N°**	**ID *A. thaliana***	**ID *Solanum tuberosum***	**Associated function**
1	*XYL1*	PGSC0003DMT400037696	Thermoinhibition resistant germination 1.
2	*PDIL2-2*	PGSC0003DMT400001769	Arabidopsis thaliana protein disulfide isomerase 10.
3	*XYL1*	PGSC0003DMT400041054	Thermoinhibition resistant germination 1.
4	*CNX1*	PGSC0003DMT400036920	Calnexin homolog 1.
5	*PDIL1-1*	PGSC0003DMT400045299	Arabidopsis thaliana protein disulfide isomerase 5.
6	*Pnsl5*	PGSC0003DMT400013412	Peptidyl-prolyl cis-trans isomerase b (cyclophilin b).
7	*PDIL1-2*	PGSC0003DMT400036388	Encodes a protein disulfide isomerase-like (PDIL) protein.
8	*CRT1b*	PGSC0003DMT400007177	Calreticulin 1b.
9	*ROC3*	PGSC0003DMT400004125	Peptidyl-prolyl cis-trans isomerase CYP19-1.
10	*FKBP12*	PGSC0003DMT400083778	Arabidopsis thaliana fk506-binding protein 12.
11	*SOS3*	PGSC0003DMT400023568	Calcium-binding EF-hand family protein.
12	*ROC3*	PGSC0003DMT400011344	Peptidyl-prolyl cis-trans isomerase CYP19-1.

Upregulated Genes Related to Mitotic cell cycle and Associated function.

**N°**	**ID *A. thaliana***	**ID *Solanum tuberosum***	**Associated function**
1	*AT3G42660*	PGSC0003DMT400063296	Transducin family protein / WD-40 repeat family protein.
2	*EMB2656*	PGSC0003DMT400034628	ARM repeat superfamily protein; EMBRYO DEFECTIVE 2656 (EMB2656).

3	*AT3G57060*	PGSC0003DMT400076712	Condensin complex subunit 1.
4	*CYCA2;4*	PGSC0003DMT400067117	Cyclin A2;4 (CYCA2;4).
5	*CYCA3;1*	PGSC0003DMT400030222	Putative cyclin-A3-1; Cyclin A3;1 (CYCA3;1).
6	*SYN3*	PGSC0003DMT400065014	Sister chromatid cohesion 1 protein 3.
7	*CYCA3;1*	PGSC0003DMT400030176	Putative cyclin-A3-1.
8	*POLA2*	PGSC0003DMT400044958	DNA polymerase alpha subunit B.
9	*AT5G52220*	PGSC0003DMT400040416	Conserved gene of unknown function.
10	*SMC3*	PGSC0003DMT400003351	Structural maintenance of chromosomes protein 4.
11	*AT4G16970*	PGSC0003DMT400067513	Protein kinase superfamily protein.
12	*CYCB2;3*	PGSC0003DMT400003868	Cyclin B2;3 (CYCB2;3).
13	*CYC1BAT*	PGSC0003DMT400015245	G2/mitotic-specific cyclin-B.
14	*CYCB2;4*	PGSC0003DMT400025854	G2/mitotic-specific cyclin-B.
15	*MCM3*	PGSC0003DMT400021420	Minichromosome maintenance (MCM2/3/5) family protein.
16	*AT1G07270*	PGSC0003DMT400078033	Cell division control protein 6.
17	*MCM6*	PGSC0003DMT400035464	Minichromosome maintenance (MCM2/3/5) family protein.
18	*CDC45*	PGSC0003DMT400071831	CDC45 (Cell division cycle 45)-like protein.
19	*AT2G20635*	PGSC0003DMT400082809	ATP binding protein kinases protein serine/threonine kinases.
20	*AUR1*	PGSC0003DMT400065470	Serine/threonine-protein kinase Aurora-1.
21	*PPK1*	PGSC0003DMT400074667	Serine/threonine-protein kinase ttk/mps1.
22	*At1g03780*	PGSC0003DMT400056261	Targeting protein for XKLP2.

23	*AT3G60660*	PGSC0003DMT400018071	Spindle and kinetochore-associated protein 1 homolog.
24	*TUB8*	PGSC0003DMT400053750	Tubulin beta-8 chain.
25	*TUB8*	PGSC0003DMT400037093	Tubulin beta-8 chain.
26	*TUB8*	PGSC0003DMT400078206	Tubulin beta-8 chain.
27	*AT5G60930*	PGSC0003DMT400024240	P-loop containing nucleoside triphosphate hydrolases superfamily protein.
28	*TUB8*	PGSC0003DMT400028800	Tubulin beta-8 chain.
29	*SGA2*	PGSC0003DMT400078369	Probable histone chaperone ASF1A.
30	*CDT1A*	PGSC0003DMT400028257	Chromatin licensing and DNA replication factor 1.
31	*BUBR1*	PGSC0003DMT400025922	BUB1-related.
32	*Haspin*	PGSC0003DMT400047135	Serine/threonine-protein kinase haspin.
33	*MAD2*	PGSC0003DMT400045025	Mitotic spindle checkpoint protein MAD2.
34	*CYCA3;2*	PGSC0003DMT400055428	Cyclin-dependent protein kinase 3;2 (CYCA3;2).
35	*AT3G02820*	PGSC0003DMT400080556	Zinc knuckle (CCHC-type) family protein.
36	*AT4G15890*	PGSC0003DMT400011993	Uncharacterized protein AT4g15890; Its function is described as binding.

Upregulated Genes Related to E2F activation and Associated function.

**N°**	**ID *A. thaliana***	**ID *Solanum tuberosum***	**Associated function**
1	*ORC6*	PGSC0003DMT400008733	Origin of replication complex subunit 6.
2	*ORC4*	PGSC0003DMT400078378	Origin recognition complex subunit 4.

3	*ORC5*	PGSC0003DMT400000500	Origin recognition complex protein 5.
4	*CDT1a*	PGSC0003DMT400028257	Chromatin licensing and DNA replication factor 1.
5	*SPT16*	PGSC0003DMT400046987	Global transcription factor C.
6	*TSO2*	PGSC0003DMT400052217	Ferritin/ribonucleotide reductase-like family protein.
7	*MCM6*	PGSC0003DMT400035464	Minichromosome maintenance (MCM/3/5) family protein.
8	*SMC3*	PGSC0003DMT400003351	Structural maintenance of chromosomes protein 4.
9	*MCM2*	PGSC0003DMT400005594	Minichromosome maintenance (MCM/3/5) family protein.
10	*RNR1*	PGSC0003DMT400060658	Ribonucleoside-diphosphate reductase large subunit.
11	*CHR11*	PGSC0003DMT400040011	ISWI chromatin-remodeling complex ATPase CHR11.
12	*POLA2*	PGSC0003DMT400044958	DNA polymerase alpha subunit B.
13	*PSF2*	PGSC0003DMT400060503	DNA replication complex GINS protein PSF2.
14	*CHR1*	PGSC0003DMT400038561	ATP-dependent DNA helicase DDM1.
15	*TOPII*	PGSC0003DMT400017488	DNA topoisomerase 2.
16	*RPA2*	PGSC0003DMT400035809	Replication protein A 32 kDa subunit A.
17	*RPA70B*	PGSC0003DMT400063329	Replication protein A 70 kDa DNA-binding subunit B.
18	*MCM5*	PGSC0003DMT400030898	Minichromosome maintenance (MCM2/3/5) family protein.
19	*RECQL1*	PGSC0003DMT400007294	ATP-dependent DNA helicase Q-like 1.
20	*ATIM*	PGSC0003DMT400016842	Timeless family protein.
21	*MCM4*	PGSC0003DMT400079535	Minichromosome maintenance (MCM/3/5) family protein.
22	*THY-1*	PGSC0003DMT400001937	Bifunctional dihydrofolate reductase-thymidylate synthase 1.

23	*PHR2*	PGSC0003DMT400081378	Photolyase/blue-light receptor 2.
24	*TIM*	PGSC0003DMT400080556	Triosephosphate isomerase, chloroplastic.
25	*SYN3*	PGSC0003DMT400065014	Sister chromatid cohesion 1 protein 3.
26	*BRCA1*	PGSC0003DMT400023253	Arabidopsis thaliana breast cancer susceptibility1.
27	*PCNA2*	PGSC0003DMT400078207	Proliferating cell nuclear antigen 2.
28	*MCM3*	PGSC0003DMT400021420	Minichromosome maintenance (MCM2/3/5) family protein.
29	*CDC6*	PGSC0003DMT400078033	Cell division control protein 6.
30	*CDC45*	PGSC0003DMT400071831	CDC45 (Cell division cycle 45)-like protein.
31	*POLA3*	PGSC0003DMT400032030	DNA primases; POLA3.

Upregulated Genes Related to Cytoskeleton and Associated function.

**N°**	**ID *A. thaliana***	**ID *Solanum tuberosum***	**Associated function**
1	*AIR9*	PGSC0003DMT400054558	Outer arm dynein light chain 1 protein.
2	*AT3G27640*	PGSC0003DMT400032878	Transducin/WD40 repeat-like superfamily protein.
3	*AT4G02110*	PGSC0003DMT400023253	BRCT domain-containing protein At4g02110.
4	*AT4G16970*	PGSC0003DMT400067513	Protein kinase superfamily protein.
5	*BUBR1*	PGSC0003DMT400025922	BUB1-related (BUB1: budding uninhibited by benzymidazol 1).
6	*TUB8*	PGSC0003DMT400053750	Tubulin beta-8 chain.
7	*Haspin*	PGSC0003DMT400047135	Serine/threonine-protein kinase haspin.
8	*TUB8*	PGSC0003DMT400078206	Tubulin beta-8 chain.

9	*TUB8*	PGSC0003DMT400037093	Tubulin beta-8 chain.
10	*EB1C*	PGSC0003DMT400064236	Microtubule-associated protein RP/EB family member 1C.
11	*TUB8*	PGSC0003DMT400028800	Tubulin beta-8 chain.
12	*AT3G60660*	PGSC0003DMT400018071	Spindle and kinetochore-associated protein 1 homolog.
13	*AUR1*	PGSC0003DMT400065470	Serine/threonine-protein kinase Aurora-1.
14	*AT5G60930*	PGSC0003DMT400024240	P-loop containing nucleoside triphosphate hydrolases super-family protein.
15	*AT2G36200*	PGSC0003DMT400023094	P-loop containing nucleoside triphosphate hydrolases superfamily protein.
16	*AT4G21820*	PGSC0003DMT400042507	Its function is described as calmodulin binding. Located in chloroplast.
17	*PPK1*	PGSC0003DMT400074667	Serine/threonine-protein kinase ttk/mps1.
18	*AT3G45850*	PGSC0003DMT400040181	P-loop containing nucleoside triphosphate hydrolases superfamily protein.
19	*MAP65-1*	PGSC0003DMT400033471	65-kDa microtubule-associated protein 1.
20	*PLE*	PGSC0003DMT400070431	Microtubule associated protein (MAP65/ASE1) family protein.
21	*AT3G45850*	PGSC0003DMT400005133	P-loop containing nucleoside triphosphate hydrolases superfamily protein.
22	*MAP65-5*	PGSC0003DMT400008510	Microtubule-associated protein 65-5.
23	*At1g03780*	PGSC0003DMT400056261	Targeting protein for XKLP2.
24	*MAP65-5*	PGSC0003DMT400057428	Microtubule-associated protein 65-5.
25	*AT3G45850*	PGSC0003DMT400078142	P-loop containing nucleoside triphosphate hydrolases superfamily protein.

Upregulated Genes Related to Motor proteins and Associated function.

N°	ID *A. thaliana*	ID *Solanum tuberosum*	Associated function
1	*AT3G45850*	PGSC0003DMT400078142	P-loop containing nucleoside triphosphate hydrolases superfamily protein.
2	*AT5G60930*	PGSC0003DMT400024240	P-loop containing nucleoside triphosphate hydrolases superfamily protein.
3	*AT2G36200*	PGSC0003DMT400023094	P-loop containing nucleoside triphosphate hydrolases superfamily protein.
4	*AT3G45850*	PGSC0003DMT400040181	P-loop containing nucleoside triphosphate hydrolases superfamily protein.
5	*AT3G45850*	PGSC0003DMT400005133	P-loop containing nucleoside triphosphate hydrolases superfamily protein.

Upregulated Genes Related to Methylation and Associated function.

N°	ID *A. thaliana*	ID *Solanum tuberosum*	Associated function
1	*UPM1*	PGSC0003DMT400065013	Uroporphyrin-iii c-methyltransferase.
2	*AT4G03130*	PGSC0003DMT400056558	BRCT domain-containing DNA repair protein.
3	*AT2G41450*	PGSC0003DMT400033151	N-acetyltransferases; Its function is described as N-acetyltransferase activity.
4	*VIM1*	PGSC0003DMT400084297	Zinc finger (C3HC4-type RING finger) family protein.
5	*MET1*	PGSC0003DMT400079464	DNA (cytosine-5)-methyltransferase 1.
6	*CMT3*	PGSC0003DMT400010941	DNA (cytosine-5)-methyltransferase CMT3.
7	*CYP51G1*	PGSC0003DMT400042257	Sterol 14alpha-demethylase. Involved in sterol biosynthesis.
8	*SMT1*	PGSC0003DMT400017668	Cycloartenol-C-24-methyltransferase.

9	*APG1*	PGSC0003DMT400049690	S-adenosyl-L-methionine-dependent methyltransferases superfamily protein.
10	*G-TMT*	PGSC0003DMT400006168	Tocopherol O-methyltransferase, chloroplastic.
11	*SMT2*	PGSC0003DMT400047969	24-methylenesterol C-methyltransferase 2.
12	*PMEAMT*	PGSC0003DMT400014749	S-adenosyl-L-methionine-dependent methyltransferases superfamily protein.
13	*THY-1*	PGSC0003DMT400001937	Bifunctional dihydrofolate reductase-thymidylate synthase 1.

Upregulated Genes Related to Histone binding and Associated function.

N°	ID *A. thaliana*	ID *Solanum tuberosum*	Associated function
1	*AT3G57060*	PGSC0003DMT400056558	Condensin complex subunit 1.
2	*SGA2*	PGSC0003DMT400078369	Probable histone chaperone ASF1A.
3	*SPT16*	PGSC0003DMT400046987	Global transcription factor C; Component of the FACT complex, a general chromatin factor that acts to reorganize nucleosomes.
4	*VIM1*	PGSC0003DMT400084297	Zinc finger (C3HC4-type RING finger) family protein.
5	*AT4G15890*	PGSC0003DMT400011993	Uncharacterized protein AT4g15890.

Upregulated Genes Related to Sterol biosynthetic process and terpenoid backbone biosynthesis and Associated function.

N°	ID *A. thaliana*	ID *Solanum tuberosum*	Associated function
1	*APG1*	PGSC0003DMT400049690	S-adenosyl-L-methionine-dependent methyltransferases superfamily protein.

2	*G-TMT*	PGSC0003DMT400006168	Tocopherol O-methyltransferase, chloroplastic.
3	*VTE5*	PGSC0003DMT400043567	Phytol kinase 1, chloroplastic.
4	*BAS*	PGSC0003DMT400048796	Terpenoid cyclases family protein; Converts oxidosqualene to beta-amyrin.
5	*RTNLB19*	PGSC0003DMT400018853	Plant 3beta-hydroxysteroid-4alpha-carboxylate 3-dehydrogenase.
6	*CYP51G1*	PGSC0003DMT400042257	Sterol 14alpha-demethylase.
7	*SMT1*	PGSC0003DMT400017668	Cycloartenol-C-24-methyltransferase.
8	*DWF1*	PGSC0003DMT400054476	Cell elongation protein / DWARF1 / DIMINUTO (DIM).
9	*HYD1*	PGSC0003DMT400071184	Probable 3-beta-hydroxysteroid-Delta(8), Delta(7)-isomerase.
10	*FK*	PGSC0003DMT400007043l	Ergosterol biosynthesis ERG4/ERG24 family.
11	*SMT2*	PGSC0003DMT400047969	24-methylenesterol C-methyltransferase 2.
12	*CPI1*	PGSC0003DMT400011907	Cycloeucalenol cycloisomerase.
13	*SMO1-1*	PGSC0003DMT400033236	Plant 4,4-dimethylsterol c-4alpha-methyl-monooxygenase.
14	*STE1*	PGSC0003DMT400067881	Delta(7)-sterol-C5(6)-desaturase 1.
15	*AT4G00560*	PGSC0003DMT400000064	NAD(P)-binding Rossmann-fold superfamily protein.
16	*SMO2-1*	PGSC0003DMT400005519	Sterol 4-alpha-methyl oxidase 1

Upregulated Genes Related to Amino sugar and nucleotide sugar metabolism and Associated function.

**N°**	**ID *A. thaliana***	**ID *Solanum tuberosum***	**Associated function**
1	*CYT1*	PGSC0003DMT400022961	Glucose-1-phosphate adenylyltransferase family protein.
2	*GME*	PGSC0003DMT400050094	GDP-D-mannose 3',5'-epimerase.
3	*UGD2*	PGSC0003DMT400087395	UDP-glucose 6-dehydrogenase family protein.
4	*RHM1*	PGSC0003DMT400018192	Rhamnose biosynthesis 1.
5	*NRS/ER*	PGSC0003DMT400058533	UDP-4-KETO-6-DEOXY-D-GLUCOSE-3,5-EPIMERASE-4-REDUCTASE 1.
6	*UGP2*	PGSC0003DMT400034699	UTP-glucose-1-phosphate uridylyltransferase 1.
7	*UGD2*	PGSC0003DMT400003666	UDP-glucose 6-dehydrogenase family protein.

Upregulated Genes Related to Sulfur metabolism and Associated function.

**N°**	**ID *A. thaliana***	**ID *Solanum tuberosum***	**Associated function**
1	*APS1*	PGSC0003DMT400048741	3'-phosphoadenosine 5'-phosphosulfate synthase.
2	*APK*	PGSC0003DMT400055243	ADENOSINE-5'-PHOSPHOSULFATE (APS) KINASE 1.
3	*HL*	PGSC0003DMT400022081	3'(2'), 5'-bisphosphate nucleotidase.
4	*APR1*	PGSC0003DMT400042488	5'-adenylylsulfate reductase 1, chloroplastic.
5	*APR3*	PGSC0003DMT400051168	5'-adenylylsulfate reductase 3, chloroplastic.
6	*CYSC1*	PGSC0003DMT400000156	Bifunctional L-3-cyanoalanine synthase/ cysteine synthase C1.

7	*SERAT2;2*	PGSC0003DMT400037270	Serine acetyltransferase 3, mitochondrial.
8	*OASA1*	PGSC0003DMT400082113	O-acetylserine (thiol) lyase (oas-tl) isoform a1.

Upregulated Genes Related to Disulfide isomerase activity and Associated function.

**N°**	**ID *A. thaliana***	**ID *Solanum tuberosum***	**Associated function**
1	*AT3G42660*	PGSC0003DMT400063296	Transducin family protein / WD-40 repeat family protein.
2	*CNX1*	PGSC0003DMT400036920	Calnexin homolog 1.
3	*PDIL1-1*	PGSC0003DMT400045299	Arabidopsis thaliana protein disulfide isomerase 5.
4	*CRT1b*	PGSC0003DMT400007177	Calreticulin 1b.
5	*PDIL1-2*	PGSC0003DMT400036388	Encodes a protein disulfide isomerase-like (PDIL) protein, a member of a multigene family within the thioredoxin (TRX) superfamily.

Upregulated Genes Related to DNA replication and Associated function

**N°**	**ID *A. thaliana***	**ID *Solanum tuberosum***	**Associated function**
1	*PDIL2-2*	PGSC0003DMT400001769	Arabidopsis thaliana protein disulfide isomerase 10.
2	*RNR1*	PGSC0003DMT400060658	Ribonucleoside-diphosphate reductase large subunit.
3	*RECQL1*	PGSC0003DMT400007294	ATP-dependent DNA helicase Q-like 1.
4	*ETG1*	PGSC0003DMT400062461	Mini-chromosome maintenance complex-binding protein.
5	*PCNA2*	PGSC0003DMT400078207	Proliferating cell nuclear antigen 2.

6	*AT4G18590*	PGSC0003DMT400022900	Nucleic acid-binding, OB-fold-like protein.
7	*TSO2*	PGSC0003DMT400052217	Ferritin/ribonucleotide reductase-like family protein.
8	*RPA2*	PGSC0003DMT400035809	Replication protein A 32 kDa subunit A.
9	*AT3G02820*	PGSC0003DMT400080556	Zinc knuckle (CCHC-type) family protein.
10	*AT4G16970*	PGSC0003DMT400067513	Protein kinase superfamily protein.
11	*AT1G07270*	PGSC0003DMT400078033	Cell division control protein 6.
12	*POLA2*	PGSC0003DMT400044958	DNA polymerase alpha subunit B.
13	*MCM3*	PGSC0003DMT400021420	Minichromosome maintenance (MCM2/3/5) family protein.
14	*DPB2*	PGSC0003DMT400079711	Dna polymerase epsilon subunit 2.
15	*MCM10*	PGSC0003DMT400015838	Minichromosome maintenance protein 10.
16	*POLA3*	PGSC0003DMT400032030	DNA primases.
17	*MCM6*	PGSC0003DMT400035464	Minichromosome maintenance (mcm2/3/5) family protein.
18	*RPA70B*	PGSC0003DMT400063329	Replication protein A 70 kDa DNA-binding subunit B.
19	*CDC45*	PGSC0003DMT400071831	CDC45 (Cell division cycle 45)-like protein.
20	*MCM4*	PGSC0003DMT400079535	Minichromosome maintenance (mcm2/3/5) family protein.
21	*SPT16*	PGSC0003DMT400046987	Global transcription factor C.
22	*AT3G27640*	PGSC0003DMT400032878	Transducin/WD40 repeat-like superfamily protein.
23	*ORC5*	PGSC0003DMT400000500	Origin recognition complex protein 5.
24	*ORC6*	PGSC0003DMT400008733	Origin of replication complex subunit 6.
25	*CDT1A*	PGSC0003DMT400028257	Chromatin licensing and DNA replication factor 1.

26	*ORC4*	PGSC0003DMT400078378	Origin recognition complex subunit 4.
27	*MCM5*	PGSC0003DMT400030898	Minichromosome maintenance (MCM2/3/5) family protein.
28	*MCM2*	PGSC0003DMT400005594	Minichromosome maintenance (mcm2/3/5) family protein.
29	*PSF2*	PGSC0003DMT400060503	DNA replication complex GINS protein PSF2.
30	*PRL*	PGSC0003DMT400023333	Minichromosome maintenance (MCM2/3/5) family protein.
31	*AT3G55490*	PGSC0003DMT400078521	Dna replication complex gins protein psf3-like.
32	*DEL1*	PGSC0003DMT400062857	E2F transcription factor-like E2FE.

Primer design of DEG used to validate the genome-wide analysis of MTs development of potato *S. tuberosum* cv. Alpha.

Gen ID	Sequences	ID NCBI
UBQ1	L: GCTTCGTGGAGGGATTATTGA	XM_006339693.1
	R: GAGGATGTAGGCGAGCATAAC	
FKBP12	L: GGGAGTGGAGAAGGAAGTTATC	XM_006351679.2
	R: GGTCTCTATCTTTCCCGTAACC	
RPL29	L: AATCAGTCGTACAAGGCTCAC	XM_015312492.1
	R: GCATACCTCTGGTTCCTCAAG	
RPL11	L: GAGAAGCTGCCGGATCTTAAT	XM_006343698.2
	R: TCGGAGGGTCAATGTCAATTC	
PRXQ	L: ATGGCTTCTCTATCCGTCTCT	XM_006353712.2
	R: CTGGAGACAATGGTGAGGTTT	
PCNA2	L: CTACGTCTTGTTCAGGGAAGTC	XM_006350753.2
	R: ATGGCTTGCAGAGAGAATCC	
SWI/SNF	L: GTGTTGCTGCACCTGTAAATC	XM_006356044.2
	R: CACCGAGTGGTGAATGTAGAA	
TSO2	L: ACCTAACATCCGGTGAAAGAC	XM_006338637.2
	R: GGCGATTTGGAACCCATAGA	
BRCA1	L: CGGTGTGCAAAGTTCCATTTC	XM_006354922.2
	R: TTGACTCCTGAAGCGATTTCC	
RNR1	L: GAGTAACCGTGACGAGTGTATG	XM_006346669.2
	R: CAGCAGGCATGGAGAGATAAA	
THY-1	L: GTGCTAAGGTCCTACAAGAGAAG	XM_006365063.2
	R: CCAAATCACCCTCTTCCCTATC	
E2F3	L: CCTGAGGATTACCACCGATTT	XM_006348424.2
	R: ATAGCCTGTCTTCCGCTTTAG	
LOG10	L: GGCGGGTTCAAGATAGGTAAA	XM_006346105.2
	R: CTACATCTCACAACAGCATCCA	

EFα1	L: TTTGGCCCTACTGGTTTGAC	NM_001288491.1
	R: GCACTGGAGCATATCCGTTT	
SEC3	L: GCTTGCACACGCCATATCAAT	XM_006342542.2
	R: TGGATTTTACCACCTTCCGCA	

References

1. Chen, G.-H.; Liu, M.-J.; Xiong, Y.; Sheen, J.; Wu, S.-H. TOR and RPS6 Transmit Light Signals to Enhance Protein Translation in Deetiolating *Arabidopsis* Seedlings. *Proc. Natl. Acad. Sci. U.S.A.* **2018**, *115* (50), 12823–12828. https://doi.org/10.1073/pnas.1809526115.
2. Kim, Y.-K.; Kim, S.; Shin, Y.; Hur, Y.-S.; Kim, W.-Y.; Lee, M.-S.; Cheon, C.-I.; Verma, D. P. S. Ribosomal Protein S6, a Target of Rapamycin, Is Involved in the Regulation of RRNA Genes by Possible Epigenetic Changes in Arabidopsis. *Journal of Biological Chemistry* **2014**, *289* (7), 3901–3912. https://doi.org/10.1074/jbc.M113.515015.
3. Tzafrir, I. The Arabidopsis SeedGenes Project. *Nucleic Acids Research* **2003**, *31* (1), 90–93. https://doi.org/10.1093/nar/gkg028.
4. Wang, R.; Zhao, J.; Jia, M.; Xu, N.; Liang, S.; Shao, J.; Qi, Y.; Liu, X.; An, L.; Yu, F. Balance between Cytosolic and Chloroplast Translation Affects Leaf Variegation. *Plant Physiol.* **2018**, *176* (1), 804–818. https://doi.org/10.1104/pp.17.00673.
5. Creff, A.; Sormani, R.; Desnos, T. The Two Arabidopsis RPS6 Genes, Encoding for Cytoplasmic Ribosomal Proteins S6, Are Functionally Equivalent. *Plant Mol Biol* **2010**, *73* (4–5), 533–546. https://doi.org/10.1007/s11103-010-9639-y.
6. Sun, L.; Yu, Y.; Hu, W.; Min, Q.; Kang, H.; Li, Y.; Hong, Y.; Wang, X.; Hong, Y. Ribosomal Protein S6 Kinase1 Coordinates with TOR-Raptor2 to Regulate Thylakoid Membrane Biosynthesis in Rice. *Biochimica et Biophysica Acta (BBA) - Molecular and Cell Biology of Lipids* **2016**, *1861* (7), 639–649. https://doi.org/10.1016/j.bbalip.2016.04.009.
7. Kim, K.-Y. Molecular Cloning of Low-Temperature-Inducible Ribosomal Proteins from Soybean. *Journal of Experimental Botany* **2004**, *55* (399), 1153–1155. https://doi.org/10.1093/jxb/erh125.
8. Ito, T.; Kim, G.-T.; Shinozaki, K. Disruption of an Arabidopsis Cytoplasmic Ribosomal Protein S13-Homologous Gene by Transposon-Mediated Mutagenesis Causes Aberrant Growth and Development. *Plant J* **2000**, *22* (3), 257–264. https://doi.org/10.1046/j.1365-313x.2000.00728.x.
9. Romani, I.; Tadini, L.; Rossi, F.; Masiero, S.; Pribil, M.; Jahns, P.; Kater, M.; Leister, D.; Pesaresi, P. Versatile Roles of Arabidopsis Plastid Ribosomal Proteins in Plant Growth and Development: *Different Functions of Plastid Ribosome Subunits*. *The Plant Journal* **2012**, *72* (6), 922–934. https://doi.org/10.1111/tpj.12000.
10. Szick-Miranda, K.; Zanial, A. S.; Zanial, A. S.; Abidayo, S.; Slater, K. L. C. Analysis of RPS15aE, an Isoform of a Plant-Specific Evolutionarily Distinct Ribosomal Protein in Arabidopsis Thaliana, Reveals Its Potential Role as a Growth Regulator. *Plant Mol Biol Rep* **2010**, *28* (2), 239–252. https://doi.org/10.1007/s11105-009-0148-6.
11. Lu, C.; Xie, Z.; Yu, F.; Tian, L.; Hao, X.; Wang, X.; Chen, L.; Li, D. Mitochondrial Ribosomal Protein S9M Is Involved in Male Gametogenesis and Seed Development in *Arabidopsis*. *Plant Biol J* **2020**, *22* (4), 655–667. https://doi.org/10.1111/plb.13108.
12. Ma, Z.; Dooner, H. K. A Mutation in the Nuclear-Encoded Plastid Ribosomal Protein S9 Leads to Early Embryo Lethality in Maize. *The Plant Journal* **2004**, *37* (1), 92–103. https://doi.org/10.1046/j.1365-313X.2003.01942.x.

13. Qiu, Z.; Chen, D.; He, L.; Zhang, S.; Yang, Z.; Zhang, Y.; Wang, Z.; Ren, D.; Qian, Q.; Guo, L.; Zhu, L. The Rice White Green Leaf 2 Gene Causes Defects in Chloroplast Development and Affects the Plastid Ribosomal Protein S9. *Rice* **2018**, *11* (1), 39. https://doi.org/10.1186/s12284-018-0233-2.
14. Tzafrir, I.; Pena-Muralla, R.; Dickerman, A.; Berg, M.; Rogers, R.; Hutchens, S.; Sweeney, T. C.; McElver, J.; Aux, G.; Patton, D.; Meinke, D. Identification of Genes Required for Embryo Development in Arabidopsis. *Plant Physiology* **2004**, *135* (3), 1206–1220. https://doi.org/10.1104/pp.104.045179.
15. Schultes, N. P.; Sawers, R. J. H.; Brutnell, T. P.; Krueger, R. W. Maize High Chlorophyll Fluorescent 60 Mutation Is Caused by an Ac Disruption of the Gene Encoding the Chloroplast Ribosomal Small Subunit Protein 17. *Plant J* **2000**, *21* (4), 317–327. https://doi.org/10.1046/j.1365-313x.2000.00676.x.
16. Chan, Y. L.; Paz, V.; Olvera, J.; Wool, I. G. The Primary Structure of L37 - a Rat Ribosomal Protein with a Zinc Finger-like Motif. *Biochemical and Biophysical Research Communications* **1993**, *192* (2), 590–596. https://doi.org/10.1006/bbrc.1993.1456.
17. Finkelshtein, A.; Khamesa, H.; Tuan, L. A.; Rabanim, M.; Chamovitz, D. A. Overexpression of the Ribosomal S30 Subunit Leads to Indole-3-carbinol Tolerance in *Arabidopsis Thaliana*. *Plant J* **2021**, *105* (3), 668–677. https://doi.org/10.1111/tpj.15062.
18. Nagaraj, S.; Senthil-Kumar, M.; Ramu, V. S.; Wang, K.; Mysore, K. S. Plant Ribosomal Proteins, RPL12 and RPL19, Play a Role in Nonhost Disease Resistance against Bacterial Pathogens. *Front. Plant Sci.* **2016**, *6*. https://doi.org/10.3389/fpls.2015.01192.
19. Zhao, D.; Zhang, C.; Li, Q.; Yang, Q.; Gu, M.; Liu, Q. A Residue Substitution in the Plastid Ribosomal Protein L12/AL1 Produces Defective Plastid Ribosome and Causes Early Seedling Lethality in Rice. *Plant Mol Biol* **2016**, *91* (1–2), 161–177. https://doi.org/10.1007/s11103-016-0453-z.
20. Yang, L.; Xie, C.; Li, W.; Zhang, R.; Jue, D.; Yang, Q. Expression of a Wild Eggplant Ribosomal Protein L13a in Potato Enhances Resistance to Verticillium Dahliae. *Plant Cell Tiss Organ Cult* **2013**, *115* (3), 329–340. https://doi.org/10.1007/s11240-013-0365-4.
21. Song, J.; Wei, X.; Shao, G.; Sheng, Z.; Chen, D.; Liu, C.; Jiao, G.; Xie, L.; Tang, S.; Hu, P. The Rice Nuclear Gene WLP1 Encoding a Chloroplast Ribosome L13 Protein Is Needed for Chloroplast Development in Rice Grown under Low Temperature Conditions. *Plant Mol Biol* **2014**, *84* (3), 301–314. https://doi.org/10.1007/s11103-013-0134-0.
22. Lee, J.; Jang, S.; Ryu, S.; Lee, S.; Park, J.; Lee, S.; An, G.; Park, S. K. Mutation of Plastid Ribosomal Protein L13 Results in an Albino Seedling-Lethal Phenotype in Rice. *Plant Breed. Biotech.* **2019**, *7* (4), 395–404. https://doi.org/10.9787/PBB.2019.7.4.395.
23. Zhang, H.; Luo, M.; Day, R. C.; Talbot, M. J.; Ivanova, A.; Ashton, A. R.; Chaudhury, A. M.; Macknight, R. C.; Hrmova, M.; Koltunow, A. M. Developmentally Regulated *HEART STOPPER* , a Mitochondrially Targeted L18 Ribosomal Protein Gene, Is Required for Cell Division, Differentiation, and Seed Development in *Arabidopsis*. *EXBOTJ* **2015**, *66* (19), 5867–5880. https://doi.org/10.1093/jxb/erv296.
24. Yan, H.; Chen, D.; Wang, Y.; Sun, Y.; Zhao, J.; Sun, M.; Peng, X. Ribosomal Protein L18aB Is Required for Both Male Gametophyte Function and Embryo Development in Arabidopsis. *Sci Rep* **2016**, *6* (1), 31195. https://doi.org/10.1038/srep31195.
25. Nishimura, T.; Wada, T.; Yamamoto, K. T.; Okada, K. The *Arabidopsis* STV1 Protein, Responsible for Translation Reinitiation, Is Required for Auxin-Mediated Gynoecium Patterning. *The Plant Cell* **2005**, *17* (11), 2940–2953. https://doi.org/10.1105/tpc.105.036533.

26. Li, S.; Liu, K.; Zhang, S.; Wang, X.; Rogers, K.; Ren, G.; Zhang, C.; Yu, B. STV1, a Ribosomal Protein, Binds Primary MicroRNA Transcripts to Promote Their Interaction with the Processing Complex in *Arabidopsis*. *Proc. Natl. Acad. Sci. U.S.A.* **2017**, *114* (6), 1424–1429. https://doi.org/10.1073/pnas.1613069114.
27. Park, S.-H.; Chung, M.-S.; Lee, S.; Lee, K.-H.; Kim, C. S. Loss of Ribosomal Protein L24A (RPL24A) Suppresses Proline Accumulation of Arabidopsis Thaliana Ring Zinc Finger 1 (Atrzf1) Mutant in Response to Osmotic Stress. *Biochemical and Biophysical Research Communications* **2017**, *494* (3–4), 499–503. https://doi.org/10.1016/j.bbrc.2017.10.092.
28. Szakonyi, D.; Byrne, M. E. Ribosomal Protein L27a Is Required for Growth and Patterning in Arabidopsis Thaliana: Ribosomal Protein-Mediated Patterning. *The Plant Journal* **2011**, *65* (2), 269–281. https://doi.org/10.1111/j.1365-313X.2010.04422.x.
29. Zsögön, A.; Szakonyi, D.; Shi, X.; Byrne, M. E. Ribosomal Protein RPL27a Promotes Female Gametophyte Development in a Dose-Dependent Manner. *Plant Physiology* **2014**, *165* (3), 1133–1143. https://doi.org/10.1104/pp.114.241778.
30. Oristian, D. S.; Sloofman, L. G.; Zhou, X.; Wang, L.; Farach-Carson, M. C.; Kirn-Safran, C. B. Ribosomal Protein L29/HIP Deficiency Delays Osteogenesis and Increases Fragility of Adult Bone in Mice. *J. Orthop. Res.* **2009**, *27* (1), 28–35. https://doi.org/10.1002/jor.20706.
31. Aseev, L. V.; Boni, I. V. Extraribosomal Functions of Bacterial Ribosomal Proteins. *Mol Biol* **2011**, *45* (5), 739–750. https://doi.org/10.1134/S0026893311050025.
32. Finley, D.; Bartel, B.; Varshavsky, A. The Tails of Ubiquitin Precursors Are Ribosomal Proteins Whose Fusion to Ubiquitin Facilitates Ribosome Biogenesis. *Nature* **1989**, *338* (6214), 394–401. https://doi.org/10.1038/338394a0.
33. Chen, J.-G. RACK1 Mediates Multiple Hormone Responsiveness and Developmental Processes in Arabidopsis. *Journal of Experimental Botany* **2006**, *57* (11), 2697–2708. https://doi.org/10.1093/jxb/erl035.
34. Guo, J.; Chen, J.-G. RACK1 Genes Regulate Plant Development with Unequal Genetic Redundancy in Arabidopsis. *BMC Plant Biol* **2008**, *8* (1), 108. https://doi.org/10.1186/1471-2229-8-108.
35. Cheng, Z.; Li, J.-F.; Niu, Y.; Zhang, X.-C.; Woody, O. Z.; Xiong, Y.; Djonović, S.; Millet, Y.; Bush, J.; McConkey, B. J.; Sheen, J.; Ausubel, F. M. Pathogen-Secreted Proteases Activate a Novel Plant Immune Pathway. *Nature* **2015**, *521* (7551), 213–216. https://doi.org/10.1038/nature14243.
36. Kakehi, J.-I.; Kawano, E.; Yoshimoto, K.; Cai, Q.; Imai, A.; Takahashi, T. Mutations in Ribosomal Proteins, RPL4 and RACK1, Suppress the Phenotype of a Thermospermine-Deficient Mutant of Arabidopsis Thaliana. *PLoS ONE* **2015**, *10* (1), e0117309. https://doi.org/10.1371/journal.pone.0117309.
